# Development and Validation of Sensitive, Fast and Simple LC-MS/MS Method to Investigate the Association between Adrenocortical Steroidogenesis and the High Intensity Exercise in Elite Athletes

**DOI:** 10.3390/metabo13070825

**Published:** 2023-07-05

**Authors:** Marina A. Dikunets, Grigory A. Dudko, Edward D. Virus

**Affiliations:** 1 Federal Science Center of Physical Culture and Sport, Elizavetinsky Lane 10/1, 105005 Moscow, Russia; dikunets.m.a@vniifk.ru (M.A.D.); edwardvirus@yandex.ru (E.D.V.); 2 Institute of General Pathology and Pathophysiology, Baltiyskaya St. 8, 125315 Moscow, Russia

**Keywords:** LC-MS/MS, steroid hormones, elite athletes

## Abstract

The very high intensity of exercise accompanied by mental stress triggers adaptive mechanisms associated with adrenocortical steroidogenesis. However, the association between adrenocortical steroidogenesis and the high intensity of exercise in elite athletes is poorly studied. A significant obstacle to solving this complex task is the wide range (4–5 orders) of steroid concentrations in serum and limitations related to the amount of biological samples taken from professional athletes. To solve this task, we have developed and validated a non-trivial approach for targeted serum metabolic profiling based on the use of LC-MS/MS with dual-polarity electrospray ionization. The developed method based on the proposed approach allows for the quantitative determination of 14 stress resistance biomarkers in elite athletes using a small amount of specimen within 8.5 min.

## 1. Introduction

The metabolome of circulating glucocorticoids reflects their powerful and versatile effects on carbohydrate, lipid, and protein metabolism. Their impact on protein and lipid metabolism in tissues and organs (except for the liver) is predominantly catabolic, while the effect of glucocorticoids on carbohydrate metabolism is expressed in the stimulation of gluconeogenesis. This leads to an increase in blood glucose and the accumulation of glycogen in the liver [1]. The main effect of glucocorticoids on protein metabolism is the mobilization of amino acid resources and the synthesis induction (in particular, in the liver) of several enzymes. Glucocorticoids inhibit protein synthesis in many tissues, including muscle [2]. This leads to a shift in equilibrium between the synthesis and cleavage of tissue proteins towards the dominance of the latter. As a result, there is an increase in the pool of free amino acids. Through the synthesis of the corresponding enzymes, glucocorticoids enhance the transamination of amino acids. Thereafter, glucocorticoids not only mobilize “building materials” for adaptive protein synthesis but also prepare them for their intended use [3]. In turn, the metabolome of circulating androgens reflects their general anabolic effect on the body and, in particular, their role in the synthesis of contractile proteins in skeletal muscles, which is especially intense during the recovery period after performing intensive muscle work [4]. During the training process, due to adaptive protein synthesis, a transition is achieved from an urgent adaptation of the body to physical activity to a long-term one, which is based on the morphofunctional improvement of cellular structures [5]. One of the important aspects of adaptation to chronic muscular activity is an increase in the sensitivity of a trained organism to the metabolic action of hormones, determined by the presence of cytoplasmic and nuclear receptors in target organs [6].

Intensive physical and mental loads launch adaptive mechanisms that affect the hypothalamus, hypophysis, and adrenal system of the athletes during the training periods [7,8]. It is well known that depending on the size of the training stimulus, determined by such variables as load, volume, duration, modality, and restoration, hormones mediate specific adaptive shifts [9]. Therefore, endogenous steroids are increasingly considered biomarkers of the functional state of athletes [10]. In a number of studies, the serum ratio concentration of testosterone to cortisol has been used as a bioindicator of overload [6,11,12]. Moreover, different changes in steroid concentrations have been shown in trained athletes and untrained individuals under equivalent loads. However, the association between adrenocortical steroidogenesis and exercise of high intensity in elite athletes remains poorly understood [13]. A significant obstacle to solving this complex issue is the wide range (4–5 orders) of steroid serum concentrations and limitations related to the amount of biological samples taken from highly professional athletes.

Until now, enzyme immunoassay has been largely used to determine the concentration of serum steroids and identify the association between adrenocortical steroidogenesis and high-intensity exercise in elite athletes [14,15]. Despite the obvious advantages of such a technique (high degree of automation, absence of time-consuming sample preparation), it is characterized by low specificity associated with the cross-reactivity of structurally related steroid hormones. On the other hand, the use of HPLC-MS/MS for the determination of serum steroids would overcome the aforementioned limitations. At the same time, the early developed HPLC-MS/MS methods for the determination of steroids did not take into account specific aspects related to elite athletes as subjects of the study (small volumes of samples, wide ranges of serum steroid concentrations) [16]. To the best of our knowledge, only Csöndör and colleagues used this approach to comprehensively study the association between athletes’ adrenocortical steroidogenesis and high-intensity exercise. The authors used two-dimensional ultra-performance liquid chromatography-tandem mass spectrometry with electrospray ionization [17]. The separation of 14 analytes from the isobaric compounds and lipids present in extracts was achieved by applying two-dimensional chromatography before mass spectrometric detection. The sample size and analysis time did not exceed 200 μL and 9.5 min, respectively. Although two-dimensional chromatography is a comprehensive principle, it is very complex and not familiar to most sports diagnostic laboratories.

Therefore, the aim of this work was to develop and validate a sensitive, fast, and simple LC-MS/MS method to investigate the association between adrenocortical steroidogenesis and high-intensity exercise in elite athletes. The developed procedure allows characterizing the steroidogenesis of the adrenal cortex based on the quantitative determination of 14 adrenocortical steroids using HPLC-MS/MS along with a fast, accessible, and very simple sample preparation technique. Sample preparation includes only two stages: protein precipitation and liquid-liquid extraction with a mixture of ethyl acetate:hexane (40:60 *v*/*v*).

## 2. Materials and Methods

### 2.1. Participants

Research participants included 50 males (age 23.49 ± 3.44, maximal oxygen consumption (VO_2 max_) 66.60 ± 4.47 mL/kg/min) and 35 females (age 21.88 ± 3.33 years, VO_2 max_ 59.9 ± 4.80 mL/kg/min) biathlonists, who are all members of the Russian national team, and 6 male and 4 female non-athletes (age 43.27 ± 12.82 years). All volunteers gave written permission to use their specimens for scientific purposes. The study was conducted in accordance with the Declaration of Helsinki and approved by the Ethics Committee of the Federal Science Center of Physical Culture & Sport, Moscow, Russia.

### 2.2. Blood Sampling

All volunteers arrived at the laboratory recovered and in a fasting condition. After 30 min of rest, venous blood was drawn from the basilica vein in serum tubes. A blood sample was gently mixed with a clot activator, and after 30 min on the table, tubes were centrifuged for 10 min at 3000× *g* rpm. The serum was then transferred to a polypropylene tube and processed in accordance with the experiment protocol.

### 2.3. Chemicals and Reference Standards

Steroids of high purity (≥98.0%): testosterone (T), 5α-dihydrotestosterone (DHT), cortisone (E), cortisol (F), dehydroepiandrosterone (DHEA), androstenedione (ADION), corticosterone (17-DF), 11-deoxycortisol (11-DF), 21-deoxycortisol (21-DF), 17α-hydroxyprogesterone (17-OH-P), 21-hydroxyprogesterone (21-OH-P), 17α-hydroxypregnenolone (17-OH-PREG), dehydroepiandrosterone sulfate sodium salt (DHEAS), and progesterone (P) were obtained from Sigma-Aldrich, St. Louis, MO, USA. Isotope-labeled internal standards (IS) of high chemical and isotopic purities (both ≥98.0%) for cortisone-d8 (E-d8), cortisol-d4 (F-d4), and testosterone-d3 (T-d3) were supplied by Sigma-Aldrich, St. Louis, MO, USA; Toronto Research Chemicals, Toronto, ON, Canada and Witega, Berlin, Germany, respectively. The 17α-methyltestosterone (MT) was bought from LGC Standards, London, the United Kingdom.

LC-MS-grade acetonitrile (AcN), LC-MS-grade methanol (MeOH), HPLC-grade hexane (Hex), HPLC-grade ethylacetate (EtAc), and acetic acid (glacial) were sourced from Merck, Darmstadt, Germany. The HPLC-grade pentane and LC-MS-grade formic acid (FA) were obtained from Honeywell, Seelze, Germany. Ammonium formate and ammonium acetate were purchased from Sigma-Aldrich, St. Louis, MO, USA. All the chemicals and solvents were of the highest purity available from commercial sources and used without further purification. The Zero Standard of DHT-optimized ELISA RUO (LOT 801S110-2, expiration date 30 November 2023) provided by DRG International, Springfield, NJ, USA was used as a blank sample, and aliquots were stored at −80 °C prior to use. Deionized water with a specific electroconductivity of 18.2 MΩ cm was generated by Milli-Q Integral 3, Millipore, Molsheim, France.

Then 2-mL and 1-mL 96-well microplates from NEST Biotechnology, Wuxi, China) were used.

Serum vacuum tubes with clot activator and separation gel were supplied by Greiner Bio-One, Kremsmunster, Austria.

### 2.4. Preparation of Calibration Standards

Steroid and internal standards stock solutions, as well as calibrator solutions at concentrations of 1 mg/mL, were prepared in MeOH. Quality control (QC) and calibration samples were prepared by spiking 25 μL of the respective calibrator solution in the blank sample to the concentrations given below.

Calibration curves for steroids were plotted using six calibration standards. Linearity ranges were 1–200 ng/mL for E; 5–250 ng/mL for F; 0.01–20 ng/mL for T; 0.1–2 ng/mL for DHT; 0.01–10 ng/mL for ADION; 0.1–15 ng/mL for DHEA; 0.1–5 μg/mL for DHEAS; 0.5–20 ng/mL for 21-DF; 0.5–20 ng/mL for 17-DF; 0.1–20 ng/mL for 11-DF; 0.05–10 ng/mL for 21-OH-P; 0.1–10 ng/mL for 17-OH-P; 1–50 ng/mL for 17-OH-PREG and 0.1–50 ng/mL for P.

QC samples were prepared at two levels (low QCL and high QCH): 3 and 150 ng/mL for E; 15 and 225 ng/mL for F; 0.03 and 17.5 ng/mL for T; 0.3 and 1.8 ng/mL for DHT; 0.3 and 12.5 ng/mL for DHEA; 0.03 and 7.5 ng/mL for ADION; 0.3 and 4.5 μg/mL for DHEAS; 1.5 and 17.5 ng/mL for 21-DF; 1.5 and 17.5 ng/mL for 17-DF; 0.3 and 17.5 ng/mL for 11-DF; 0.3 and 7.5 ng/mL for 17-OH-P; 0.15 and 7.5 ng/mL for 21-OH-P; 3 and 45 ng/mL for 17-OH-PREG; 0.3 and 45 ng/mL for P.

An IS working solution made up of E-d8 (250 ng/mL), F-d4 (250 ng/mL), T-d3 (500 ng/mL), and MT (250 ng/mL) was prepared in MeOH. All solutions were stored at −20 °C until analysis.

### 2.5. Sample Preparation

100 μL of specimens, calibrators, and QCs were mixed with 10 μL of IS working solution and 250 μL of ACN. After vigorous stirring for 60 s using a vortex apparatus, the mixture was centrifuged (5 min at 2000× *g* rpm) and 300 μL of supernatant was transferred into a clean tube. Then 1.8 mL of EtAc:Hex (40:60, *v*/*v*) was added, and analytes were extracted by vigorous mechanical shaking for 10 min. The tube was then centrifuged (5 min at 3000× *g* rpm), and the organic layer (1.0 mL) was transferred to a 2-mL 96-well microplate and evaporated for approximately 20 min under nitrogen flow (approximately 25 L/min) while being heated to 40 °C. The dry residue was reconstituted in 150 μL of MeOH:H2O (50:50, *v*/*v*). Finally, the microplate was sealed with a silicone mat and vortexed for 5 min at room temperature and 2000× *g* rpm, followed by centrifugation at 1666× *g*, after which 120 μL of the solution was transferred into a 1-mL 96-well microplate.

### 2.6. Instrumental Analysis

Separation was achieved on an Acquity UPLC BEH C18 column (100 × 2.1 mm, 1.7 μm, Waters Inc., Milford, MA, USA) coupled with the guard column Acquity UPLC BEH C18 (5 × 2.1 mm, 1.7 μm, Waters Inc., Milford, MA, USA). Mobile phases were aqueous at 0.1% FA (mobile phase A) and 0.1% FA in MeOH (mobile phase B). The gradient elution program was as follows: 0.0 min, 45% (B); 4.5 min, 65% (B); 6.0–7.0 min, 95% (B); 7.01–8.5 min, 45% (B). The mobile phase flow rate was set to 350 μL/min, the column oven temperature was maintained at 60 °C, and the injection volume was equal to 10 μL. The total analysis time was 8.5 min.

UPLC-MS/MS analysis was performed on an ultra-performance liquid chromatograph-triple quadrupole tandem mass spectrometer consisting of the following modules: a DGU-20A5R degassing unit, two LC-30AD pumps, a SIL-30AC autosampler, a CTO-20AC column oven, a CBM-20A system controller, an LCMS-8060 triple quadrupole mass spectrometer operated simultaneously in positive and negative electrospray ionization (ESI) modes (all—Shimadzu Corporation, Kyoto, Japan), a nitrogen and dry air generator Genius 1051, Peak Scientific Instruments Ltd. (Inchinnan, Scotland, The United Kingdom). LabSolutions software (Shimadzu Corporation, Kyoto, Japan), version 5.99, was used for instrument control, data acquisition, and processing.

Quantitative data were obtained in multiple reaction monitoring (MRM) mode from protonated [M + H]^+^ or deprotonated [M − H]^−^ precursor ions. Two specific mass transitions were chosen for all analytes except DHEAS: one for confirmation (the “qualitative transition”) and one for quantification (the “quantitative transition”). One very specific mass transition was chosen for this analyte. The MRM acquisition settings are summarized in Table 1. Interface voltage was set at +4/−4 kV, and collision-induced dissociation (CID) gas was maintained at 17 kPa. Nebulizer gas was set at 3 L/min, and drying and heating gas flows were both kept at 10 L/min. Interface, desolvation line, and heat block temperatures were 300, 250, and 400 °C, respectively.

Statistical data processing was performed using STATISTICA, version 10 (StatSoft Inc., Tulsa, OK, USA).

### 2.7. Method Validation

The method’s performance was evaluated by means of linearity, the lower limit of quantification (LLOQ), the limit of detection (LOD), intra- and inter-assay precision, accuracy, recovery, and ion suppression. Hormone stability in the matrix, stock solutions, and samples, when stored under different temperature conditions, were also assessed. Linearity was established by analyzing triply replicated calibrators at six levels. The acceptance criterion for linearity was a correlation factor (r^2^) ≥ 0.99. LLOQ was determined as the lowest measured concentration with accuracy within 80–120% of the expected value and precision expressed as a relative standard deviation, RSD < 20%. LOD was estimated as the lowest measured concentration, with a signal-to-noise ratio of 3:1. Intra-assay precision was determined by measuring each level of QC samples in six replicates (*n* = 6) within a single batch. Inter-assay precision was assessed by measuring each level of QC samples in six replicates over three consecutive days (*n* = 18). The criteria for intra- and inter-assay acceptance were RSD within ±10% and accuracy within 90–110% of nominal concentration. Extraction yield assessment was carried out on two concentration levels of analytes corresponding to those in QC samples in triplicate. In this regard, calibrator solutions were spiked in a blank sample and, along with unspiked aliquots, subjected to the current approach. The extraction yield in percentage was calculated as:

Extraction yield = [[(concentration in spiked sample − concentration in unspiked sample)/ known spiked concentration]] × 100%(1)

Ion suppression (in percentage) was evaluated following the post-extraction addition protocol by comparing the peak areas of all analytes added post-extraction in a blank sample (at 100 pg/mL) to those of a pure solution with an equivalent amount prepared in a mixture of MeOH:H_2_O (50:50, *v*/*v*). Analyte stability in the matrix was estimated using fresh QCL and QCH samples, which were analyzed immediately after preparation and then placed in the refrigerator at 2–8 °C for 24 and 72 h. Beyond these time points, stored samples were re-analyzed using calibration curves plotted from freshly prepared solutions, and the obtained concentrations of analytes were compared to nominal values. Analyte stability in stock solutions at 1 mg/mL expressed as a percentage of peak area variation was calculated by comparing peak areas of stored in the freezer (−18 to −20 °C) solutions to those freshly prepared. For this reason, model solutions were prepared by diluting fresh and stored stock solutions to 10 μg/mL with MeOH and analyzing them in six replicates. The acceptance criteria for analyte stability in stock solutions were peak area variety within ±15%. Analyte stability in samples was assessed by quantification of steroid hormones in serum aliquots from volunteers (*n* = 10), which after initial analysis were stored for: (i) 6 h at room temperature; (ii) 24 h at room temperature; (iii) 6 h at 2–8 °C; (iv) 24 h at 2–8 °C; (v) 6 weeks at –80 °C; (vi) 24 h at –20 °C (one freeze-thaw cycle); and (vii) two freeze-thaw cycles. Stability was expressed as a relative error in percentage. The statistical significance of differences between analyte stabilities in serum was checked using the Wilcoxon signed-rank test (T), the critical value of which (Tcrit) according to the tabular data for *n* = 10 at a significance level of *p* < 0.05 satisfied the inequality Tcrit > 10.

## 3. Results

All steroids except DHEAS were detected in positive MRM mode using electrospray ionization. The mass spectra of steroids obtained via direct injection of standards into an ionization source were used to search for optimal MS/MS spectra and select an adequate product ion for quantification. A protonated molecule observed in all spectra of steroids except DHEAS was chosen as the precursor ion. For this analyte, a deprotonated molecule [M-H]^–^ was chosen as the precursor ion. For the generation of MS/MS spectra, precursor ions were fragmented under optimized parameters (Table 1).

**Figure 1:**
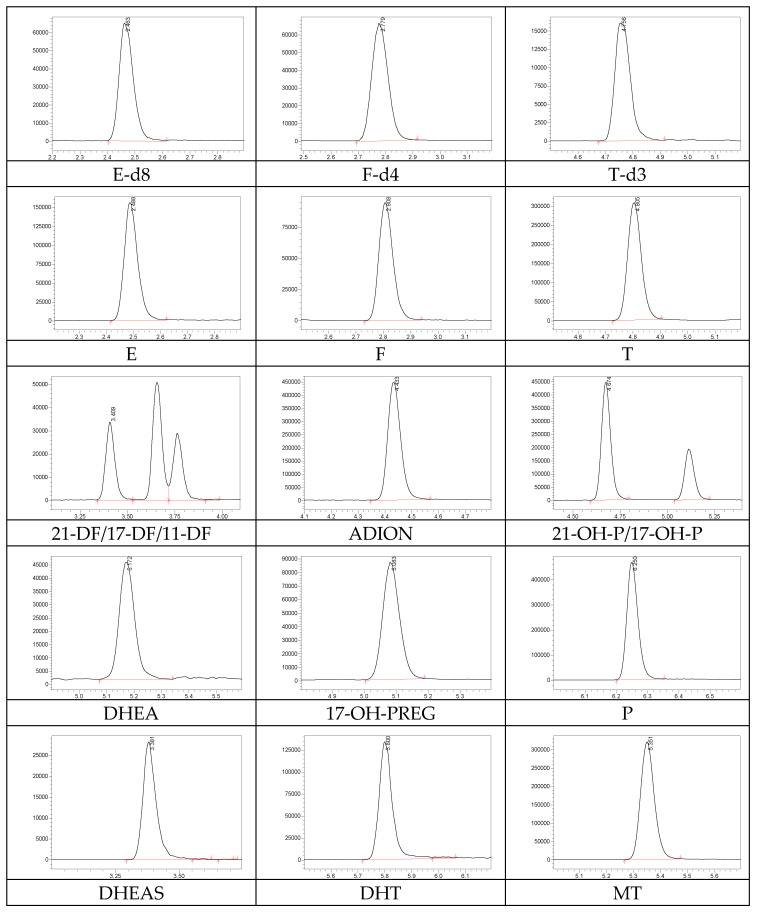
Multiple reaction monitoring chromatograms of analytes in the stock solution.

The matching of optimal chromatographic conditions for the separation of steroid hormones consisted of testing the mobile phase (AcN, MeOH), modifier (formic acid, acetic acid, ammonium formate, and ammonium acetate), and chromatographic column (Cortecs UPLC C18, Acquity UPLC BEH C18). In accordance with early studies, the greatest efficiency of ionization for all analytes was achieved using formic acid as a modifier. The signal for all studied steroids significantly increased when 0.1% formic acid was used. Moreover, the best separation of 14 analytes was achieved using the Acquity UPLC BEH C18 analytical column with formic acid as a modifier (Figure 1). Chromatographic peaks were symmetric, the critical peaks were resolved at 10% of their respective peak heights, and all analytes eluted between 2.4 and 6.4 min.

**Figure 2:**
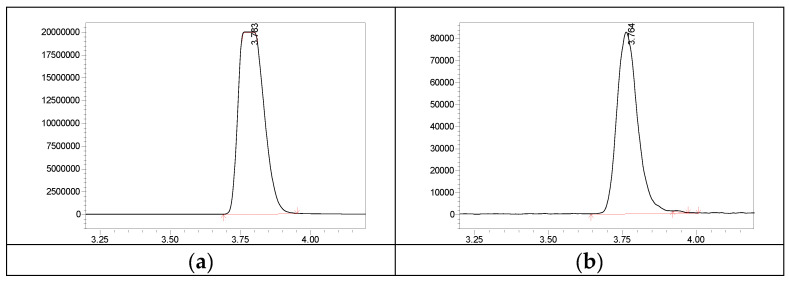
Multiple reaction monitoring chromatograms of DHEAS extracted from human serum with EtAc (**a**) and the EtAc:Hex mixture (**b**).

For the optimal extraction of the selected steroid panel, diethyl ether, pentane, Hex, and EtAc were tested as extractants. The extraction yield for all studied steroids is shown in Table 2. For all steroids studied, the extraction yield was above 50% except DHEAS when using the EtAc:Hex mixture. Regardless of the fact that DHEAS recovery was equal to 0.4%, it was enough for quantitative determination. The use of a mixture of EtAc:Hex (40:60 *v*/*v*) made it possible to simultaneously avoid detector saturation and achieve an acceptable extraction yield for DHEAS (Figure 2b).

To establish the validation characteristics of the developed assay, linearity, LOD, LLOQ, intra- and inter-day precision, accuracy (Table 2), and ion suppression factor (Table 3) were determined. The calibration curve was calculated with the least-squares method. The calibration curves showed good linearity in the range of 0.25–5.000 ng/mL for all analytes (Table 2). The correlation coefficients (r^2^) of the calibration curves were higher than 0.992 (Table 2), and the LOD and LLOQ were 0.01–1 (Table 3) and 0.015–5 ng/mL (Table 3), respectively. The calibration data are summarized in Table 2. As shown in Table 3, the precision and accuracy of the method were determined by analyzing QC samples at different concentrations (low, 0.075–150 ng/mL and high, 1.25–4500 ng/mL) of the individual steroids. Intra-day (*n* = 6) precisions [expressed as (relative standard deviation) RSD] ranged from 3.03% to 6.97%, whereas accuracies (expressed as percentage bias) ranged from 92.78% to 97.94%, and inter-day (*n* = 18) precisions and accuracies ranged from 4.21% to 8.44% and from 91.06% to 97.02%, respectively (Table 3). The ion suppression factor (*n* = 3) varied from 84.44% to 97.61%. These results indicate that the ion suppression did not significantly influence method reliability (Table 3).

In the case of the impossibility of performing LC-MS/MS immediately after blood sample collection, we evaluated the stability of steroid hormones in serum under different temperatures and time conditions (Table 4). Stock solutions of steroids were stored at −20 °C and remained stable throughout the whole experiment. The serum steroid concentrations measured in athletes’ samples, expressed as mean ± standard deviation (SD), are summarized in Table 5.

## 4. Discussion

**Figure 3:**
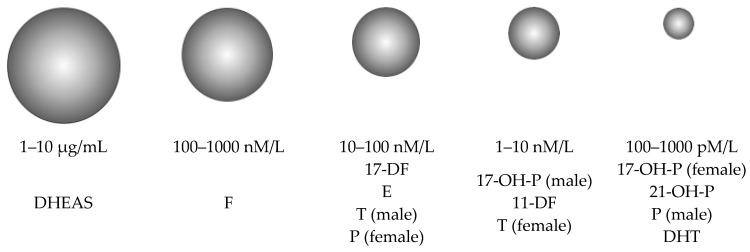
Graphical representation of circulating serum steroid hormone concentrations [18].

Assay development for 14 serum steroid hormones in simultaneous quantitation represented a non-trivial analytic challenge since they circulate in the bloodstream in a wide range of concentrations, conventionally divided into five ranges. A graphical presentation of the serum steroid metabolome illustrating their relative contribution to the total circulating steroid pool is shown in Figure 3. Therefore, we were faced with the issue of developing a steroid hormone simultaneous quantitation approach that should ensure the linear dependence of the analytical signal on the concentration of each of several analytes in individual physiological ranges of serum levels, some of which differ by 4–5 orders, e.g., DHT vs. DHEAS.

**Figure 4:**
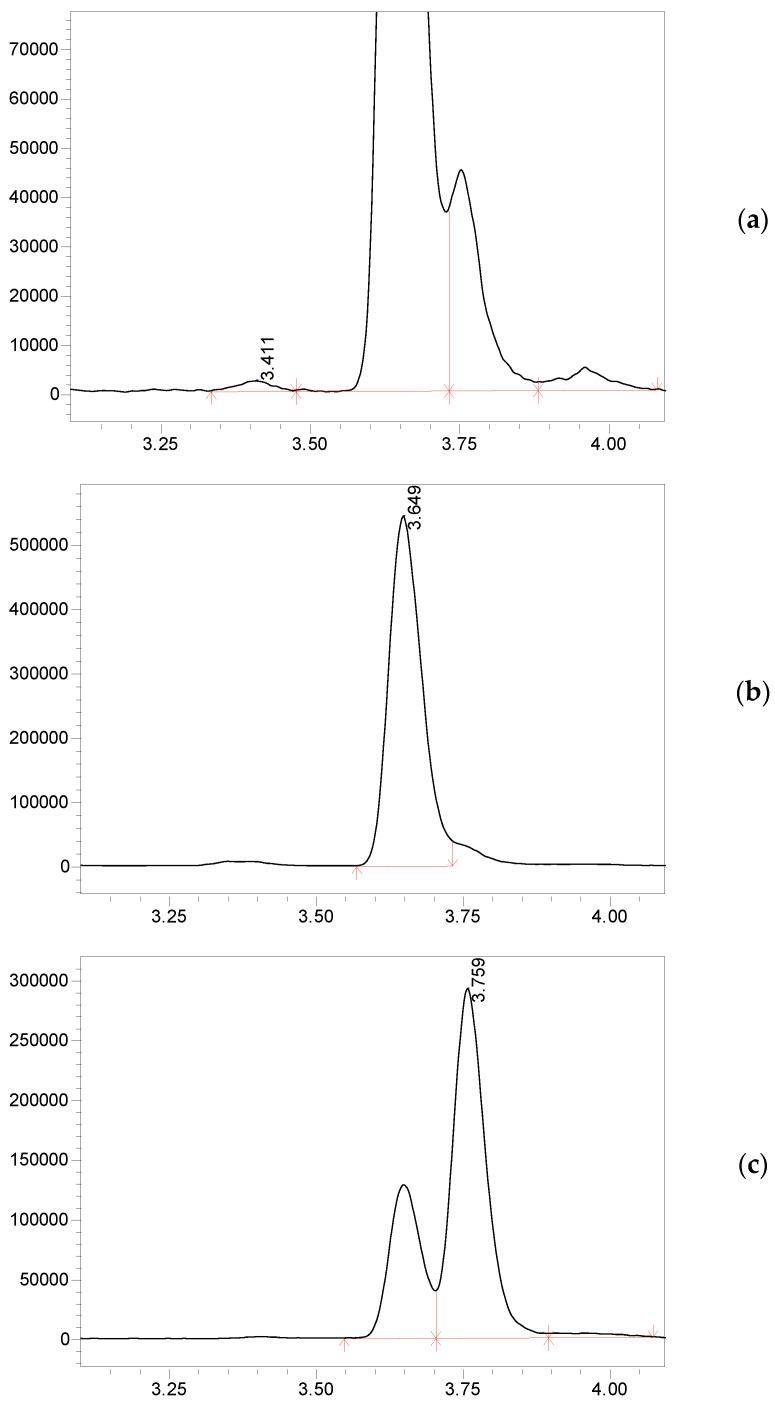
LS-MS/MS chromatograms of a serum sample show the separation of 11-DF, 17-DF, and 21-DF, which share similar fragmentation patterns and retention times. (**a**) 21-DF, *m*/*z* 347.1 → 121.1; (**b**) 17-DF, *m*/*z* 347.1 → 121.1; (**c**) 11-DF, 347.1 → 109.2.

The similar fragmentation pattern for 11-DF, 17-DF, and 21-DF in ESI conditions complicates their MS/MS detection. The above-mentioned steroids have similar structures and the same molecular weight. In the present work, the necessary selectivity was achieved owing to optimized chromatographic conditions that provided the separation of this critical trio in serum samples (Figure 4).

The effect of the most commonly used modifiers (formic acid, ammonium acetate, and ammonium formate) added to the mobile phase to increase the efficiency of compound ionization was also assessed within this study. On the one hand, ammonium formate increased the ionization of most analytes by ~1.5 times except DHT, which circulated in the bloodstream in the lowest concentration ranges, and DHEA. On the other hand, DHEAS did not ionize when neither ammonium formate nor ammonium acetate was added to the mobile phase. The maximum and optimal ionization of all steroid hormones was observed when using formic acid as a modifier.

Until now, liquid-liquid extraction has remained the simplest and most accessible sample preparation technique. In the present study, it was chosen as the most familiar method for sports diagnostic laboratories. The selection of tested solvents used as extractants were dictated by the properties of steroids, which, except for DHEAS, are relatively non-polar molecules. However, none of the non-polar solvents tested (diethyl ether, pentane, and Hex) were able to extract DHEAS from serum. The high extraction yield for all selected steroids from serum was achieved using EtAc (Table 1). At the same time, a high DHEAS concentration in serum leads to detector saturation and complicates quantification when extracted with EtAc (Figure 2a). Despite the fact that the extraction yield for all studied steroids was sufficiently high (Table 1) when using EtAc, we declined it on account of the detector saturation by DHEAS. Thus, a mixture of EtAc:Hex (40:60 *v*/*v*) was chosen as the optimal extractant (Figure 2b).

The protocol to assess analyte stability in serum was developed to simulate temperature conditions and storage duration of specimens depending on the time required to overcome issues obstructing the ability to perform the analysis immediately after blood collection. Analyte stability in serum was established when stored at different temperatures and storage times (Table 4). The majority of the steroid hormones that were not stable at room temperature were stored for 24 h in the refrigerator and frozen and thawed more than once. Therefore, whenever it is not possible to perform LC-MS/MS analysis straight after blood sampling, serum tubes should be stored in short-term outlook storage for no more than 6 h at 2–8 °C or 24 h in the freezer, whereas for long-term storage, specimens should be placed in a deep freeze at –80 °C. The applicability of the developed and validated LC-MS/MS method was confirmed by determining the steroids in serum samples obtained from elite biathlonists. Serum steroid levels in all subjects were observed to be significantly above the LLOQ (Table 5).

In summary, the results indicated that the current method had ascendant analytical performance and was stable and reliable under our experimental conditions.

## 5. Conclusions

A simple, sensitive, rapid, and robust HPLC-MS/MS method using liquid-liquid extraction as the sample preparation technique was developed and validated to investigate the association between adrenocortical steroidogenesis and high-intensity exercise in elite athletes. The well-designed gradient elution provided a satisfactory separation of all the analytes. The sample size did not exceed 100 μL. The proposed approach can be recommended for testing athletes during competitive periods.

## Figures and Tables

**Table 1:** MS/MS parameters for the targeted steroids and their internal standards.

Steroid		ISTD	Mode	Precursor, *m*/*z*	Product, *m*/*z*	RT, min	Dwell Time, msec	Q1 Pre Bias, V	CE, eV	Q3 Pre Bias, V
E-d8	ISTD	1	+	369.1	169.2	2.43	30	−11	−25	−30
F-d4	ISTD	2	+	367.2	121.1		30	−11	−25	−12
		3			313.2				−19	−21
MT	ISTD	4	+	303.1	109.2	5.28	30	−32	−27	−18
T-d3	ISTD	5	+	292.6	97.2	4.73	30	−12	−22	−18
		6			109.2		30		−24	−20
E	quan	1	+	361.1	163.1	2.45	30	−14	−25	−32
	qual	1			121.1		30		−31	−22
F	quan	2	+	363.1	121.1	2.76	30	−14	−23	−24
	qual	3			309.2		30		−17	−22
21-DF	quan	2	+	347.1	121.1	3.35	30	−26	−25	−22
	qual	3			105.1		30		−45	−40
17-DF	quan	2			121.1	3.59	30	−26	−25	−22
	qual	3			109.2		30		−28	−12
11-DF	quan	2			109.2	3.71	30	−26	−28	−12
	qual	3			105.1		30		−45	−40
DHEAS	quan	4	–	367.2	97.0	3.81	30	14	34	10
ADION	quan	2	+	287.1	97.1	4.36	30	−20	−22	−18
	qual	3			109.1		30		−24	−20
21-OH-P	quan	6	+	331.1	109.2	4.49	30	−12	−27	−20
	qual	5			97.1		30		−27	−18
T	quan	5	+	289.1	97.1	4.74	30	−20	−22	−18
	qual	6			109.2		30		−26	−20
17-OH-P	quan	6	+	331.1	109.2	5.03	30	−12	−27	−20
	qual	5			97.1		30		−27	−18
17-OH-PREG	quan	3	+	297.1	91.2	5.03	30	−12	−52	−16
	qual	3			105.1		30		−36	−18
DHEA	quan	4	+	271.1	253.2	5.11	30	−10	−14	−26
	qual	4			105.2		30		−37	−20
DHT	quan	5	+	291.3	255.3	5.74	30	−15	−16	−29
	qual	6			159.1		30		−22	−26
P	quan	6	+	315.1	109.2	6.21	30	−12	−25	−20
	qual	5			97.1		30		−23	−18

**Table 2:** Results of the extraction yield, ion suppression factor, linearity, and calibration curve.

Steroid	Calibration Curve Formula	r	R^2^	Linearity, ng/mL	Concentration of Calibrators, ng/mL	Ion Supression Factor, %	Extraction Yield, %	
							EtAc	MIX
E	y = 6.75 × 10^−2^X + 2.32 10^−3^	0.999	0.999	1–200	3	84.44 ± 4.95	53.71 ± 1.60	40.23 ± 3.15
					150	87.85 ± 2.44	52.43 ± 2.20	42.14 ± 3.38
F	y = 3.83 × 10^−2^X + 0.04	0.999	0.999	5–250	15	88.46 ± 4.17	53.78 ± 1.24	35.09 ± 5.27
					225	89.76 ± 2.30	53.51 ± 2.46	38.40 ± 5.20
T	y = 1.51 × X + 0.08	0.998	0.996	0.01–20	0.03	95.71 ± 3.95	63.88 ± 1.22	55.25 ± 8.77
					17.5	97.61 ± 2.79	66.01 ± 1.63	57.48 ± 4.95
DHT	y = 5.67 × 10^−5^X + 4.29 10^−3^	0.998	0.995	0.025–1.5	0.075	90.06 ± 3.18	63.61 ± 5.34	60.27 ± 5.25
					1.25	91.27 ± 2.78	65.28 ± 4.78	61.04 ± 2.28
ADION	y = 0.37 × X + 8.29 10^−3^	0.998	0.997	0.01–10	0.03	92.79 ± 8.32	68.61 ± 4.43	58.26 ± 16.71
					7.5	95.68 ± 6.17	63.73 ± 1.13	59.63 ± 4.96
DHEA	y = 7.11 × 10^−3^X + 2.76 10^−3^	0.997	0.995	0.1–15	0.3	87.50 ± 4.20	79.18 ± 1.87	72.24 ± 4.91
					12.5	89.05 ± 2.68	83.01 ± 2.97	75.69 ± 1.54
DHEAS	y = 8.84 × 10^−3^X + 1.98 10^−4^	0.997	0.995	50–5000	150	88.58 ± 3.58	61.61 ± 2.47	0.44 ± 0.03
					4500	95.17 ± 1.46	68.33 ± 2.70	0.45 ± 0.02
21-DF	y = 2.31 × 10^−2^X + 1.31 10^−3^	0.999	0.999	0.5–20	1.5	91.29 ± 4.01	52.59 ± 1.23	51.26 ± 3.93
					17.5	92.22 ± 3.70	54.14 ± 3.31	53.73 ± 6.79
17-DF	y = 5.45 × 10^−2^X + 2.81 10^−3^	0.999	0.998	0.5–20	1.5	84.35 ± 4.13	51.05 ± 1.80	49.65 ± 3.36
					17.5	87.65 ± 2.91	51.74 ± 3.59	53.41 ± 4.94
11-DF	y = 0.13 × X + 1.10 10^−3^	0.999	0.999	0.1–20	0.3	86.43 ± 2.13	54.33 ± 1.70	53.24 ± 3.99
					17.5	89.63 ± 5.02	53.39 ± 3.28	55.16 ± 7.05
21-OH-P	y = 0.16 × X + 1.05 10^−3^	0.996	0.992	0.05–10	0.15	89.89 ± 6.17	58.87 ± 5.82	58.87 ± 4.76
					7.5	91.38 ± 1.75	61.72 ± 1.73	64.79 ± 4.27
17-OHP	y = 0.14 × X + 2.62 10^−4^	0.998	0.997	0.1–10	0.3	88.15 ± 4.95	53.55 ± 1.79	58.27 ± 3.70
					7.5	94.10 ± 2.84	55.48 ± 1.28	64.89 ± 4.61
17-OH-PREG	y = 1.37 × 10^−3^X + 1.24 10^−3^	0.998	0.997	1–50	3	88.47 ± 6.21	59.37 ± 5.17	60.93 ± 2.45
					45	94.09 ± 4.16	61.71 ± 1.52	61.41 ± 6.01
P	y = 0.03 × X + 2.22 10^−3^	0.999	0.999	0.1–50	0.3	89.68 ± 4.98	56.89 ± 2.80	58.08 ± 8.38
					45	90.96 ± 3.68	62.39 ± 1.61	64.80 ± 8.67

**Table 3:** LLOQ, LOD, Inter- and Intra-day precision and accuracy for steroid quantification.

Steroid	Concentration in Calibrators, ng/mL	Precision, %		Accuracy, %		LLOQ, ng/mL	LOD, ng/mL
		Intra-Day (*n* = 6)	Inter-Day (*n* = 18)	Intra-Day (*n* = 6)	Inter-Day (*n* = 18)		
E	3	5.31	6.16	92.27	95.91	0.5	0.1
	150	5.04	5.52	95.80	96.64		
F	15	4.16	4.78	93.39	94.96	1	0.1
	225	3.63	3.95	94.71	95.22		
T	0.03	7.11	8.44	93.03	94.26	0.01	0.005
	17.5	6.39	6.12	95.82	96.14		
DHT	0.075	5.12	6.89	92.88	94.31	0.015	0.01
	1.25	4.73	5.48	94.54	94.59		
ADION	0.03	6.52	7.13	91.96	96.18	0.01	0.005
	7.5	5.08	5.54	96.37	96.81		
DHEA	0.3	4.46	5.70	95.50	95.88	0.1	0.05
	12.5	4.22	4.83	96.18	96.08		
DHEAS	150	6.97	7.67	91.06	92.78	5	1
	4500	6.01	6.63	93.18	94.69		
21-DF	1.5	3.03	5.33	95.18	96.80	0.25	0.1
	17.5	3.67	4.12	96.98	97.36		
17-DF	1.5	4.03	4.56	95.36	96.17	0.25	0.1
	17.5	3.89	4.11	97.02	97.84		
11-DF	0.3	5.48	6.64	93.94	95.12	0.1	0.05
	17.5	4.20	4.55	96.76	97.94		
21-OH-P	0.15	5.06	5.30	92.11	94.84	0.03	0.01
	7.5	4.37	4.89	96.13	94.63		
17-OH-P	0.3	5.27	5.82	94.83	95.96	0.05	0.01
	7.5	4.33	4.69	96.59	96.66		
17-OH-PREG	3	4.79	5.18	95.36	96.53	0.5	0.25
	45	4.38	4.65	96.21	96.87		
P	0.3	5.72	5.38	94.94	95.48	0.05	0.025
	45	4.03	4.21	95.46	95.98		

**Table 4:** Stability of steroids assay in human serum.

Analyte	Stability, %						
	6 h at RT ^1^	24 h at RT	6 h at 2–8 °C	24 h at 2–8 °C	6 Weeks at –80 °C	24 h at –20 °C	2 Freeze-Thaw Cycles
E	0.95 ± 1.37 (*p* = 0.355)	1.30 ± 1.95 * (*p* < 0.05)	0.69 ± 2.78 (*p* = 0.444)	1.77 ± 4.27 (*p* = 0.202)	0.17 ± 1.95 (*p* = 0.734)	0.07 ± 2.65 (*p* = 0.878)	0.13 ± 3.33 (*p* = 0.831)
F	−1.55 ± 3.48 (*p* = 0.475)	−1.87 ± 5.57 (*p* = 0.575)	−1.27 ± 4.32 (*p* = 0.575)	−1.32 ± 2.29 (*p* = 0.307)	−1.07 ± 1.37 (*p* = 0.621)	−1.36 ± 1.79 (*p* = 0.332)	−3.22 ± 2.92 * (*p* < 0.05)
T	6.39 ± 7.74 * (*p* < 0.05)	77.74 ± 11.65 * (*p* < 0.05)	0.41 ± 1.62 (*p* = 0.374)	64.64 ± 20.22 * (*p* < 0.05)	1.76 ± 1.19 (*p* = 0.237)	1.87 ± 1.24 (*p* = 0.196)	8.49 ± 5.88 * (*p* < 0.05)
DHT	3.24 ± 1.66 (*p* = 0.104)	12.91 ± 7.37 * (*p* < 0.05)	0.89 ± 0.64 (*p* = 0.575)	8.94 ± 1.37 * (*p* < 0.05)	0.15 ± 1.44 (*p* = 0.309)	0.05 ± 4.39 (*p* = 0.332)	1.76 ± 3.62 (*p* = 0.878)
ADION	6.27 ± 2.79 * (*p*<0.05)	29.89 ± 18.95 * (*p* < 0.05)	1.47 ± 2.66 (*p* = 0.541)	16.14 ± 11.43 * (*p* < 0.05)	2.21 ± 1.54 (*p* = 0.293)	2.48 ± 3.90 (*p* = 0.201)	32.37 ± 11.89 * (*p* < 0.05)
DHEA	−7.08 ± 3.97 * (*p* < 0.05)	−12.70 ± 6.54 * (*p* < 0.05)	−0.94 ± 1.07 (*p* = 0.638)	−1.74 ± 2.38 (*p* = 0.444)	0.97 ± 1.78 (*p* = 0.627)	1.10 ± 1.38 (*p* = 0.436)	10.56 ± 9.98 * (*p* < 0.05)
DHEAS	10.88 ± 6.75 * (*p* < 0.05)	27.34 ± 11.04 * (*p* < 0.05)	4.93 ± 2.53 (*p* = 0.752)	5.63 ± 3.70 (*p* = 0.505)	1.34 ± 3.49 (*p* = 0.832)	1.65 ± 4.22 (*p* = 0.752)	7.32 ± 4.09 (*p* = 0.352)
21-DF	−3.32 ± 1.90 (*p* = 0.203)	6.22 ± 1.14 * (*p* < 0.05)	−1.40 ± 1.88 (*p* = 0.452)	2.33 ± 2.57 (*p* = 0.264)	3.51 ± 3.77 (*p* = 0.347)	−1.23 ± 2.16 (*p* = 0.247)	−4.55 ± 2.32 * (*p* < 0.05)
17-DF	−2.28 ± 2.37 (*p* = 0.107)	−5.14 ± 3.79 * (*p* < 0.05)	−0.98 ± 2.54 (*p* = 0.333)	−1.98 ± 2.38 (*p* = 0.153)	−1.93 ± 2.46 (*p* = 0.178)	−2.71 ± 3.00 (*p* = 0.083)	−3.37 ± 4.67 * (*p* < 0.05)
11-DF	1.34 ± 2.46 (*p* = 0.289)	1.80 ± 3.45 (*p* = 0.258)	0.16 ± 1.96 (*p* = 0.562)	1.41 ± 2.26 (*p* = 0.362)	0.09 ± 1.74 (*p* = 0.627)	0.06 ± 2.51 (*p* = 0.528)	0.41 ± 2.18 (*p* = 0.352)
21-OH-P	20.99 ± 31.62 * (*p* < 0.05)	70.63 ± 21.36 * (*p* < 0.05)	1.43 ± 4.52 (*p* = 0.506)	21.90 ± 16.88 * (*p* < 0.05)	1.04 ± 3.61 (*p* = 0.539)	1.24 ± 5.24 (*p* = 0.453)	72.12 ± 20.45 * (*p* < 0.05)
17-OH-P	5.37 ± 4.25 * (*p* < 0.05)	9.92 ± 12.27 * (*p* < 0.05)	0.46 ± 1.29 (*p* = 0.724)	5.69 ± 4.79 * (*p* < 0.05)	0.59 ± 1.46 (*p* = 0.536)	0.25 ± 1.64 (*p* = 0.936)	4.59 ± 3.78 (*p* = 0.113)
17-OH-PREG	−7.36 ± 4.84 * (*p* < 0.05)	−12.49 ± 4.26 * (*p* < 0.05)	−0.96 ± 1.67 (*p* = 0.952)	−4.78 ± 2.23 * (*p* < 0.05)	1.92 ± 1.48 (*p* = 0.793)	2.37 ± 1.84 (*p* = 0.414)	12.94 ± 5.36 * (*p* < 0.05)
P	0.66 ± 0.51 (*p* = 0.476)	1.20 ± 2.35 (*p* = 0.260)	0.35 ± 0.59 (*p* = 0.593)	1.42 ± 2.36 (*p* = 0.285)	0.05 ± 1.33 (*p* = 0.858)	0.01 ± 2.59 (*p* = 0.764)	0.20 ± 5.20 (*p* = 0.593)

^1^ RT—room temperature; *—analyte is not stable under current conditions.

**Table 5:** The results of steroid quantification in elite athletes’ serum samples.

Analyte	Concentrations	
	Male (*n* = 50)	Female (*n* = 35)
E, ng/mL	20.76 *±* 3.94	22.13 *±* 6.91
F, ng/mL	94.74 *±* 30.27	96.40 *±* 36.74
T, ng/mL	5.51 *±* 1.78	0.40 *±* 0.18
DHT, ng/mL	0.49 *±* 0.24	0.21 *±* 0.13
ADION, ng/mL	0.55 *±* 0.18	1.03 *±* 0.42
DHEA, ng/mL	4.03 *±* 1.77	5.59 *±* 3.07
DHEAS, µg/mL	3.73 *±* 2.20	3.10 *±* 2.03
21-DF, ng/mL	0.02 *±* 0.03	0.05 *±* 0.04
17-DF, ng/mL	3.06 *±* 2.91	2.59 *±* 1.77
11-DF, ng/mL	0.22 *±* 0.21	0.22 *±* 0.18
21-OH-P, ng/mL	0.02 *±* 0.02	0.03 *±* 0.02
17-OH-P, ng/mL	0.75 *±* 0.28	0.83 *±* 0.67
17-OH-PREG, ng/mL	3.34 *±* 4.13	4.30 *±* 9.40
P, ng/mL	0.03 *±* 0.02	2.55 *±* 4.23

## Data Availability

The data presented in this study are available in the article.

## References

[1] Giordano R., Guaraldi F., Berardelli R., Karamouzis I., D’Angelo V., Marinazzo E., Picu A., Ghigo E., Arvat E. (2012). Glucose metabolism in patients with subclinical Cushing’s syndrome. Endocrine.

[2] Viru A., Viru M. (2004). Cortisol-essential adaptation hormone in exercise. Int. J. Sport. Med..

[3] Hill E.E., Zack E., Battaglini C., Viru M., Viru A., Hackney A.C. (2008). Exercise and circulating cortisol levels: The intensity threshold effect. J. Endocrinol. Investig..

[4] Kelly D.M., Jones T.H. (2013). Testosterone: A metabolic hormone in health and disease. J. Endocrinol..

[5] Hooper D.R., Kraemer W.J., Focht B.C., Volek J.S., DuPont W.H., Caldwell L.K., Maresh C.M. (2017). Endocrinological roles for testosterone in resistance exercise responses and adaptations. Sport. Med..

[6] Cook C.J., Crewther B.T., Kilduff L.P., Agnew L.L., Fourie P., Serpell B.G. (2021). Testosterone and dihydrotestosterone changes in male and female athletes relative to training status. Int. J. Sport. Physiol. Perform..

[7] Kraemer W.J., Ratamess N.A. (2005). Hormonal responses and adaptations to resistance exercise and training. Sport. Med..

[8] Cadegiani F.A., Kater C.E. (2017). Hypothalamic-pituitary-adrenal (HPA) axis functioning in overtraining syndrome: Findings from endocrine and metabolic responses on overtraining syndrome (EROS)–EROS–HPA axis. Sport. Med. Open.

[9] Saidi K., Abderrahman A.B., Hackney A.C., Bideau B., Zouita S., Granacher U., Zouhal H. (2021). Hematology, hormones, inflammation, and muscle damage in elite and professional soccer players: A systematic review with implications for exercise. Sport. Med..

[10] Kuikman M.A., Coates A.M., Burr J.F. (2022). Markers of low energy availability in overreached athletes: A systematic review and meta-analysis. Sport. Med..

[11] Tsunekawa K., Ushiki K., Martha L., Nakazawa A., Hasegawa R., Simizu R., Shimoda N., Murata M., Yoshida A., Nakajima K. (2021). Automated measurements of salivary cortisol levels within circadian rhythms detect differences in exercise-induced stress response between two altitude training camps. Res. Sq..

[12] Sato K., Iemitsu M., Katayama K., Ishida K., Kanao Y., Saito M. (2016). Responses of sex steroid hormones to different intensities of exercise in endurance athletes. Exp. Physiol..

[13] Tarkhan A.H., Anwardeen N.R., Sellami M., Donati F., Botre F., de la Torre X., Elrayess M.A. (2022). Comparing metabolic profiles between female endurance athletes and non-athletes reveals differences in androgen and corticosteroid levels. J. Steroid Biochem. Mol. Biol..

[14] Popovic B., Popovic D., Macut D., Antic I.B., Isailovic T., Ognjanovic S., Bogavac T., Kovacevic V.E., Ilic D., Petrovic M. (2019). Acute response to endurance exercise stress: Focus on catabolic/anabolic interplay between cortisol testosterone, and sex hormone binding globulin in professional athletes. J. Med. Biochem..

[15] Jimenez M., Alvero-Cruz J.R., Solla J., Garcia-Bastida J., Garcia-Coll V., Rivilla I., Ruiz E., Garcia-Romero J., Carnero E.A., Clemento-Suarez V.J. (2020). Competition seriousness and competition level modulate testosterone and cortisol responses in soccer players. Int. J. Environ. Res. Public Health.

[16] Fanelli F., Belluomo I., Di Lallo V.D., Cuomo G., De Iasio R., Baccini M., Casadio E., Casetta B., Vicennati V., Gambineri A. (2011). Serum steroid profiling by isotopic dilution-liquid chromatography-mass spectrometry: Comparison with current immunoassays and reference intervals in healthy adults. Steroids.

[17] Csondor E., Karvaly G., Ligetvari R., Kovacs K., Komka Z., Mora A., Stromajer-Racz T., Olah A., Toth M., Acs P. (2022). Adrenal, gonadal and peripherally steroid changes in response to extreme physical stress for characterizing load capacity in athletes. Metabolites.

[18] Schiffer L., Barnard L., Baranowski E.S., Gilligan L.C., Taylor A.E., Arlt W., Shackelton C.H.L., Storbeck K.H. (2019). Human steroid biosynthesis, metabolism and excretion are differentially reflected by serum and urine steroid metabolomes: A comprehensive review. J. Steroid Biochem. Mol. Biol..

